# Comment on “The association between the Kidney Donor Profile Index and one-year outcomes in Brazilian kidney transplant recipients of standard criteria donors”

**DOI:** 10.1590/2175-8239-JBN-2025-0255en

**Published:** 2025-10-17

**Authors:** Umaima Parveen, Eisha Abid

**Affiliations:** 1 Jinnah Sindh Medical University, Karachi, Pakistan.

Dear Editor,

We read with great interest the recent article by Ana Paula Aquino de Morais and colleagues entitled “The association between the Kidney Donor Profile Index and one-year outcomes in Brazilian kidney transplant recipients of standard criteria donors”^1^. The authors present an important perspective on donor risk assessment, offering findings that are both timely and clinically relevant. We commend their contribution to this evolving area of transplantation research and would like to share some observations that may complement and expand upon their work.

Firstly, the Brazilian study utilized the 1-year estimated glomerular filtration rate (eGFR) at one year as a surrogate for long-term graft survival to evaluate kidney transplant success, but this approach is limited. Having a satisfactory eGFR at one year does not guarantee sustained function in subsequent years, as kidney function can vary significantly over time, particularly in higher risk donor organ recipients, such as those with high Kidney Donor Profile Index (KDPI) scores. Long-term tracking of kidney function trajectories yields a more accurate prediction of transplant outcomes than a single one-year measurement. Additionally, the study by Coemans et al. directly demonstrated that, in comparison to eGFR alone, death-censored graft survival is more accurate and significant for assessing graft outcomes over time^2^.

Secondly, the authors used multivariable logistic regression with variables that had a p-value of less than 0.10 in the univariate analysis^1^. Although this method is frequently used, it may leave out clinically important confounders (such as age, sex, baseline eGFR, dialysis duration, and acute rejection) that are necessary for robust adjustment but do not reach statistical significance in univariate testing. In contrast, Pullerits et al. identified clinically relevant variables prior to analysis, listed them explicitly, and included them in multivariable logistic and Cox proportional hazards models^3^. Validity and generalizability can be improved by applying adjusted time-to-event models for endpoints like graft loss or mortality and by including predefined confounders. The evidence base in transplantation studies may be strengthened if such methodologies are adopted in subsequent research.

Lastly, from 2013 to 2017, the study used the pp65 antigenemia assay to track CMV reactivation. Despite the later implementation of PCR testing, antigenemia results were still used to guide clinical decisions throughout the study^1^. The use of two distinct assays over time introduces temporal inconsistency in data collection and treatment thresholds. In addition, antigenemia has lower sensitivity, especially in leukopenic patients. These factors make this approach labor-intensive and scientifically limited. As a comparison, a prospective study used quantitative CMV-PCR from the beginning for all patients, applying standardized, reproducible viral load thresholds for therapy and supplementing these with QuantiFERON-CMV to assess cellular immunity^4^. This PCR-based approach ensures earlier detection, consistent and objective clinical decisions, and reliable risk stratification advantages that are not fully achievable with antigen-based monitoring.

Overall, the authors’ findings add valuable insight to the field, and we believe that refining outcome measures, applying comprehensive adjustment for key confounders, and ensuring methodological consistency will help guide and strengthen future research in kidney transplantation.

## Data Availability

No new data were generated or analyzed in support of this article.
