# Supplementary material for: Comment on “The association between the Kidney Donor Profile Index and one-year outcomes in Brazilian kidney transplant recipients of standard criteria donors”
Source: J Bras Nefrol. 2025 Oct 17;47(4):e20250255. doi: 10.1590/2175-8239-JBN-2025-0255en (PMC12700439; doi:10.1590/2175-8239-JBN-2025-0255en)
Supplement: Translation

# Translation: Comentário sobre “Associação entre o Kidney Donor Profile Index e os desfechos em um ano em receptores de transplante renal com doadores de critério padrão no Brasil”

**DOI:** 10.1590/2175-8239-JBN-2025-0255pt

**Authors:** Umaima Parveen, Eisha Abid

**Affiliations:** 1 Jinnah Sindh Medical University, Karachi, Pakistan.

Caro Editor,

Lemos com grande interesse o recente artigo de Ana Paula Aquino de Morais e colaboradores, intitulado “Associação entre o *Kidney Donor Profile Index* e os desfechos em um ano em receptores de transplante renal com doadores de critério padrão no Brasil”^1^. Os autores apresentam uma perspectiva importante sobre a avaliação de risco do doador, oferecendo achados oportunos e clinicamente relevantes. Parabenizamos os autores por sua contribuição nessa área em evolução da pesquisa sobre transplantes e gostaríamos de compartilhar algumas observações que podem complementar e expandir seu trabalho.

Em primeiro lugar, o estudo brasileiro utilizou a taxa de filtração glomerular estimada (TFGe) em um ano como substituto para a sobrevida do enxerto em longo prazo na avaliação do sucesso do transplante renal. No entanto, essa abordagem apresenta limitações. Ter uma TFGe satisfatória em um ano não garante uma função sustentada nos anos subsequentes, uma vez que a função renal pode variar significativamente ao longo do tempo, especialmente em receptores de órgãos de doadores de maior risco, como aqueles com escores elevados no *Kidney Donor Profile Index* (KDPI). O acompanhamento a longo prazo das trajetórias da função renal fornece uma predição mais precisa dos desfechos do transplante do que uma única medição anual. Além disso, o estudo de Coemans et al. demonstrou diretamente que, em comparação com a TFGe isolada, a sobrevida do enxerto censurada para óbito é mais precisa e significativa para avaliar os desfechos do enxerto ao longo do tempo^2^.

Em segundo lugar, os autores utilizaram regressão logística multivariada com variáveis que apresentaram um valor de p inferior a 0,10 na análise univariada^1^. Embora esse método seja frequentemente empregado, ele pode não considerar fatores de confusão clinicamente relevantes (como idade, sexo, TFGe basal, tempo de diálise e rejeição aguda) que são necessários para um ajuste robusto, mas não atingem significância estatística em testes univariados. Em contraste, Pullerits et al. identificaram variáveis clinicamente relevantes previamente à análise, listaram-nas de maneira explícita e as incluíram em modelos de regressão logística multivariada e de riscos proporcionais de Cox^3^. A validade e a generalização podem ser aprimoradas pela aplicação de modelos ajustados de tempo até o evento para desfechos como perda do enxerto ou mortalidade e pela inclusão de fatores de confusão predefinidos. A base de evidências em estudos de transplante pode ser fortalecida se tais metodologias forem adotadas em pesquisas subsequentes.

Por fim, de 2013 a 2017, o estudo utilizou o ensaio de antigenemia pp65 para rastrear a reativação do CMV. Apesar da posterior implementação do teste PCR, os resultados da antigenemia continuaram a ser empregados a fim de orientar as decisões clínicas ao longo do estudo^1^. O uso de dois ensaios distintos ao longo do tempo introduz inconsistência temporal na coleta de dados e nos limites de tratamento. Além disso, a antigenemia apresenta menor sensibilidade, especialmente em pacientes leucopênicos. Esses fatores tornam essa abordagem trabalhosa e cientificamente limitada. A título de comparação, um estudo prospectivo utilizou PCR quantitativo para CMV desde o início em todos os pacientes, aplicando limites padronizados e reproduzíveis de carga viral para a terapia e complementando-os com QuantiFERON-CMV para avaliar a imunidade celular^4^. Essa abordagem baseada em PCR garante uma detecção mais precoce, decisões clínicas consistentes e objetivas, além de vantagens confiáveis na estratificação de risco, que não são totalmente alcançáveis com o monitoramento baseado em antígenos.

De modo geral, os achados dos autores acrescentam informações valiosas ao campo, e acreditamos que o refinamento das medidas de desfecho, a aplicação de ajustes abrangentes para os principais fatores de confusão e a garantia de consistência metodológica contribuirão para orientar e fortalecer pesquisas futuras no transplante renal.

## Data Availability

Nenhum dado novo foi gerado ou analisado para fundamentar este artigo.
